# Interleukin-32 positive immune and resident cells in kidney samples from lupus patients: a pilot study

**DOI:** 10.3389/fimmu.2024.1475073

**Published:** 2025-01-06

**Authors:** Simona Truglia, Francesco Ciccia, Silvia Mancuso, Antonella Capozzi, Aroldo Rizzo, Francesca Romana Spinelli, Fulvia Ceccarelli, Tania Colasanti, Cristina Garufi, Francesca Miranda, Maurizio Sorice, Cristiano Alessandri, Fabrizio Conti

**Affiliations:** ^1^ Rheumatology Unit, Department of Clinical Internal, Anesthesiologic and Cardiovascular Sciences, “Sapienza” University of Rome, Rome, Italy; ^2^ Rheumatology Unit, Department of Internal Medicine and Medical Specialties, Azienda Ospedaliera Universitaria (AOU) Policlinico Umberto I, Rome, Italy; ^3^ Department of Precision Medicine, University of Campania Luigi Vanvitelli, Naples, Italy; ^4^ Department of Experimental Medicine, “Sapienza” University of Rome, Rome, Italy; ^5^ Pathology Section, Azienda Ospedaliera Ospedali Riuniti Villa Sofia Cervello, Palermo, Italy; ^6^ Rheumatology Unit, Azienda Sanitaria Locale (ASL) Roma1, Rome, Italy

**Keywords:** lupus nephritis, toll like receptor 3, interleukin-32, lupus nephritis IgG, resident renal cells

## Abstract

**Introduction:**

Lupus nephritis (LN), caused by immune complexes produced *in situ* or deposited from the bloodstream, is one of the most severe features of Systemic Lupus Erythematosus (SLE) leading to an increased morbidity and mortality. Toll like receptors (TLRs), such as TLR3, TLR7 and TLR9, may play a key role in its pathogenesis. Interleukin-32 (IL-32), a cytokine involved in both innate and adaptive immune responses, has been widely considered in autoimmune-inflammatory rheumatic diseases. This study aims to evaluate the IL-32 role in LN, also investigating the effect of LN patients IgG (LN-IgG) on IL-32 production via TLR3.

**Methods:**

In LN patients, IL-32 was detected in sera samples by ELISA KIT and in kidney tissue by immunohistochemistry. HEK293/T3 cells were incubated with LN-IgG and analyzed for TBK1, phospho-p65 NF-κB and IL-32 by Western blot.

**Results:**

We demonstrated IL-32 presence in LN patients compared to SLE patients without renal involvement, observing a direct correlation between IL-32 serum levels and disease duration (p=0.02; r 0.2978). Moreover, IL-32 was strongly expressed in renal samples of LN patients. Phosphorylation of TBK1 resulting in NF-κB activation and IL-32 increase was observed in HEK293/T3 cells following LN-IgG treatment, TLR3 inhibitor using induced a significant reduction in the expression of these molecules.

**Discussion:**

These results showed that IL-32 is up-regulated in the kidney of LN patients suggesting that in renal tissue IL-32 expression could be induced through TLR3 activation by the LN patients’ antibodies. This study may indicate a possible role for IL-32 in the pathogenesis of LN.

## Introduction

Lupus nephritis (LN) is one of the most severe features of Systemic Lupus Erythematosus (SLE) and leads to an increased morbidity and mortality. Although the five-year mortality for LN showed a decline from 1975 to 1995, it subsequently stabilized. Additionally, the progression rate to end-stage kidney disease (ESKD) has remained stable (1, 2). Moreover, data from the LUMEN registry show that the incidence of LN from 1976 to 2018 has remained essentially unchanged, in particular mortality has not improved over the last 40 years. Furthermore, 13% of patients developed ESRD and only 61% were alive and had no ESRD or renal transplantation at 10-year follow-up (3).

LN is caused by immune complexes, produced *in situ* or deposited from the bloodstream, which activate innate and adaptive immune response (4, 5). Toll-Like Receptors (TLRs) play a key role in modulating the innate immune response recognizing molecular patterns. Among them, TLR3 binds double-stranded RNA (dsRNA), TLR7 and TLR8 bind single-stranded RNA and TLR9 binds microbial unmethylated cytidine-guanidine repeat sequences (CpG-DNA) (6, 7). In murine models (MRL/lpr), activation of TLR3 and TLR7 has been shown to contribute to the progression of renal involvement (8, 9). Additionally, TLR8 appears to play a regulatory role over TLR7 and is involved in the pathogenesis of lupus nephritis. Notably, mouse models with a TLR8 deletion develop glomerulonephritis, but this effect is absent when both TLR7 and TLR8 are deleted (10). Furthermore, the TLR9 polymorphism (rs351240) has been associated with a predisposition to developing lupus nephritis (11). In a previous study we revealed that TLR3, TLR7 and TLR9 are overexpressed in the kidney biopsy of LN patients compared with healthy controls (HC) and correlated with clinicopathological indices such as disease activity and chronicity index (12).

Recent *in vitro* studies demonstrated that the Poly I:C, a synthetic analogue of dsRNA, strongly induces Interleukin-32 (IL-32) production in epithelial cells (13) and fibroblast-like synoviocytes (14).

IL-32, previously named Natural Killer Cell Transcript 4 (NK4), is a cytokine that plays a crucial role in both innate and adaptive immune responses. It is produced by various cell types, such as monocytes, T cells, Natural Killer (NK) cells, fibroblasts, epithelial cells, and endothelial cells, in response to various inflammatory stimuli. IL-32 triggers the differentiation of monocytes into macrophages or dendritic cells and induces the production of pro-inflammatory cytokines, including tumor necrosis factor (TNF), IL-1β, IL-6, and IL-8, through the nuclear factor-kappa B (NF-κB) and p38 mitogen-activated protein (MAP) kinase inflammatory signal pathway (15–17).

Unsurprisingly, an increasing number of studies explored the involvement of IL-32 in autoimmune-inflammatory rheumatic diseases (AIRD), such as rheumatoid arthritis (RA), ankylosing spondylitis (AS), psoriatic arthritis (PsA), as well as SLE (17, 18). Two studies showed increased levels of IL‐32γ in LN patients compared to healthy controls and SLE patients without LN (19, 20). Moreover, Kwon and colleagues demonstrated an overexpression of IL-32 in renal tissue of LN patients compared to those healthy renal samples (20).

The purpose of this study is to evaluate in depth the role of IL-32 in LN by investigating serum, urinary and tissue expression of IL-32 in LN patients. We also evaluated the *in vitro* effect of IgG isolated from patients with LN on IL-32 production in the human embryonic kidney cells via TLR3.

## Materials and methods

### Patients

Consecutive patients aged >18 years who met the European League against Rheumatism/American College of Rheumatology classification criteria for SLE (21) were recruited from the Sapienza Lupus Clinic of Rome. Patients were recorded as having LN if they fulfilled one of the following renal criteria: persistent proteinuria greater than 0.5 grams per day or greater than 3+ if quantitation not performed or cellular casts (22). The LN patients were divided into two groups according to the presence (a-LN) or absence (r-LN) of LN activity based on Systemic Lupus Erythematosus Disease Activity Index SLEDAI score (23). Patients who underwent a biopsy for the evaluation of kidney involvement as a standard of care management were classified in accordance with the ISN/RPS classification criteria (24). For each patient the following variables were recorded at study entry: age, sex, ethnicity, disease duration, hypertension, smoking habits and drug therapy.

All patients and control subjects included in this study provided written informed consent. Data were used anonymously in accordance with the latest version of the Helsinki Declaration of human research ethics. The study protocol was approved by the local ethics committees of the Policlinico Umberto I, Rome, Italy.

### Sample collection

A 5-ml peripheral venous blood sample was drawn from each study participant; sera were obtained by centrifugation and stored at −20°C until the examination. A fresh urine sample (midstream) from each patient was collected in a sterile container; the urine was centrifuged to remove sediments then frozen in aliquots at −80°C for later test.

Renal tissues were obtained from LN patients and, as control, from HC at the time of kidney donation.

### Dosage of IL-32

Serum and urinary IL-32 evaluations were assessed using commercially available IL-32 DuoSet^®^ ELISA KIT (R&D systems, Minneapolis, MN, USA), according to the manufacturer’s instructions. The same kit was used for the evaluation of IL-32 in urine samples. This sandwich ELISA kit is validated for serum samples, but like other ELISA kits or multiplex immunoassays kit, specific for the dosage of cytokines, it is also used for other biological fluids such as urine, after centrifugation and urinary sediment discard (25–27).

### Immunohistochemistry and light microscopy

Paraffin-embedded sections of 5-μm thickness were stained with haematoxylin and eosin (H&E) for the histological evaluation. Immunohistochemistry was performed on 5-μm-thick paraffin-embedded sections from SG, as previously described (12). The primary antibody mouse anti-human IL-32 (Novus Biologicals, Littleton, CA) was added and incubated for 1 h at room temperature (RT). Isotype-matched irrelevant antibody was used as a negative control (Abcam, Cambridge, UK). The number of positive cells was determined by counting the reactive cells on microphotographs obtained from three randomly selected high-power microscopic fields (original magnification × 400).

### Cell cultures and treatments

Human embryonic kidney 293 (HEK293) cells, stably transfected with TLR3 gene (HEK293T3) (InvivoGen, San Diego, CA, USA) were maintained in Dulbecco’s Modified Eagle Medium (DMEM, Sigma-Aldrich, Milan, Italy), containing 50 U/ml penicillin, 50 mg/ml streptomycin, 100 mg/ml Normocin™, 2 mM L-glutamine, 10 μg/ml Blasticidin as selective antibiotic, 10% fetal calf serum (FCS, Sigma-Aldrich).

In some experiments control untransfected HEK293 were used. Cell cultures were grown under standard conditions at 37°C in a humified atmosphere containing 5% CO_2_.

Ig fractions were isolated from sera of a-LN patients or healthy donors using a slightly modified protocol involving precipitation with, as previously described (28). HEK293T3 cells were incubated, at 37°C for different incubation times, with purified IgG from LN patients (LN-IgG, 200 μg/ml), normal human serum IgG (NHS- IgG, 200 μg/ml) or 10 μg/ml Poly(I:C) (InvivoGen), a synthetic analog of dsRNA considered a potent activator of TLR3 and can therefore be used as a positive control. To investigate TLR3 involvement, cells were also treated with a TLR3 inhibitor (27 μM, TLR3/dsRNA Complex Inhibitor, Merck Millipore, Milano, Italy) alone or in combination with LN-IgG.

We preliminary determined the optimal IgG concentration and incubation time on the basis of concentration/time curve; all the experiments were shown at the best concentration and incubation time.

### Western blot analysis of phospho-TBK1 and NF-κB

To prepare whole protein extracts, HEK293T3 cells, untreated or treated with LN-IgG, NHS-IgG, Poly(I:C), or TLR3 inhibitor, for 45 min, were resuspended in RIPA lysis buffer (50 mM Tris-HCl pH 7.4, 0.5% Triton X-100, 0.25% Nadeoxycholate, 0.1% SDS, 150 mM NaCl, 1mM EDTA and 5mM MgCl_2_, including proteases and phosphatases inhibitors). Protein content was determined by Bradford assay, using bovine serum albumin (BSA) as standard (Bio-Rad, Richmond, CA, USA). Equal amounts of whole protein extracts were subjected to 10% sodium dodecyl sulfate (SDS-PAGE) and then blotted onto polyvinylidene difluoride (PVDF) membranes (Bio-Rad). After blocking with 5% defatted dried milk in Tris-buffered saline (TBS), containing 0.05% Tween-20 membranes were probed with rabbit polyclonal anti-phospho-TBK1/NAK or anti-phospho-NF-κB p65 antibodies (Cell Signaling, Inc Danvers, MA, USA). Bound antibodies were visualized with HRP-conjugated anti-rabbit IgG antibodies (Sigma-Aldrich) and immunoreactivity was assessed by the enhanced chemiluminescence (ECL) Western blotting system (Amersham Pharmacia Biotech, Piscataway, NJ, USA). To control nonspecific reactivity and verify sample loading, PVDF membranes were stripped and reprobed with anti-rabbit IgG and anti-β-actin Abs respectively (Sigma-Aldrich). Densitometric scanning analysis was performed by Mac OS X (Apple Computer International), using NIH Image 1.62 software. The density of each band in the same gel was analysed, and the densitometric TBK1/β-actin or p65/β-actin ratios are shown.

### IL-32 cell expression

Sample of HEK293T3 and HEK293 cells untreated or treated, for 16 h, with LN-IgG, NHS-IgG, Poly(I:C), or TLR3 inhibitor were lysed as described above, separated in 10% SDS-PAGE and then analysed by Western blot as previously described. Membranes were incubated with rabbit polyclonal anti-IL-32 (Abcam) followed by HRP-conjugated anti-rabbit IgG antibodies (Sigma-Aldrich). To control nonspecific reactivity and verify sample loading, PVDF membranes were stripped and reprobed with anti-rabbit IgG and anti-β-actin Abs respectively (Sigma-Aldrich. Densitometric scanning analysis was performed by Mac OS X (Apple Computer International), using NIH Image 1.62 software. The density of each band in the same gel was analysed, and the densitometric IL-32/β-actin ratios are shown.

### Statistical analysis

All the statistical analyses were performed by GraphPad Prism software Inc., (San Diego, CA, USA). Data were expressed as mean ± standard deviation (SD) or median [interquartile range (IQR)], according to the distribution. Kolmogorov-Smirnov test was used to assess the normal distribution of the data. Differences between numerical variables were evaluated with Kruskal-Wallis and Mann-Whitney tests. Correlation was tested with Pearson’s correlation coefficient in normally distributed variables or Spearman’s rank order in variables which are not normally distributed. For comparison of categorical variables or percentages we used Fisher’s exact and X^2^ tests when appropriate. For Western blot experiments, statistical analysis was performed using Student’s t test. P-values less than 0.05 were considered as significant.

## Results

### Patients characteristics and clinical manifestations

Sixty SLE patients with LN (51 females and 9 males) with a mean age of 37 years (SD 11.65), 50 SLE patients without renal involvement (43 females and 7 males) with a mean age of 45 years (SD 14.5) and 30 healthy control samples recruited from the donor transfusion center of our hospital, with age and sex carefully matched with the patients groups to ensure no significant differences. All the patients and controls were Caucasian. **Table 1** shows demographic and clinical features of SLE patients with and without renal involvement. Forty (66.7%) LN patients had an active renal disease [a-LN, median SLEDAI-2K 11.5 (3-5)] and 20 patients (33.3%) were in renal remission [r-LN, median SLEDAI-2K 0 (0-8)]. **Table 2** summarizes clinical and demographic data of LN patients.

**Table 1:** Demographic and clinical data of the Lupus patients with and without renal involvement.

	LN N= 60	SLE N= 50	*p*
Sex F/M	51/9	43/7	–
AGE (mean ± SD, years)	37.0 ± 11.65	45 ± 14.5	NS
DISEASE DURATION [median (IQR), months]	132 (6-396)	72 (1-372)	**0.002**
CREATININE (mg/dl) [median (IQR)]	1.0 (0.5-3)	1 (0.1-1)	NS
PROTEINURIA (g/24h) [median (IQR)]	537.5 (0-11385)	–	–
C3 (mg/dl) [median (IQR)]	83 (28-480)	103 (65-143)	**0.037**
C4 (mg/dl) [median (IQR)]	18 (4-190)	21 (10-41)	NS
SLEDAI-2K [median (IQR)]	8 (0-23)	0 (0-10)	**<0.001**

LN, lupus nephritis; SLE, Systemic Lupus Erythematosus; C3 and C4, complement; SLEDAI, Systemic Lupus Erythematosus Disease Activity Index.

The bold values represent statistically significant values.

**Table 2:** Demographic and clinical data of the Lupus Nephritis cohort.

	a-LN N= 40	r-LN N=20	*p*
Sex F/M	35/5	16/4	
AGE (mean ± SD, yrs)	36 ± 11.7	39 ± 11.7	NS
DISEASE DURATION [median (IQR), months]	132.0 (6-396)	144 (6-360)	**0.001**
CREATININE (mg/dl) [median (IQR)]	1.0 (0.5-3)	0.8 (0,4-2,7)	NS
PROTEINURIA (mg/24h) [median (IQR)]	1616 (330-15800)	180 (0-500)	**<0.000**
C3 (mg/dl) [median (IQR)]	73 (28-480)	99 (49-175)	**0.013**
C4 (mg/dl) [median (IQR)]	15 (4-190)	25 (5-142)	NS
SLEDAI-2K [median (IQR)]	11.5 (3-23)	0 (0-8)	**<0.000**

a-LN, active lupus nephritis; r-LN, remission lupus nephritis; C3 and C4, complement; SLEDAI, Systemic Lupus Erythematosus Disease Activity Index. The bold values represent statistically significant values.

### Serum and urinary level of IL-32 in LN patients and clinical correlations

**Figure 1:**
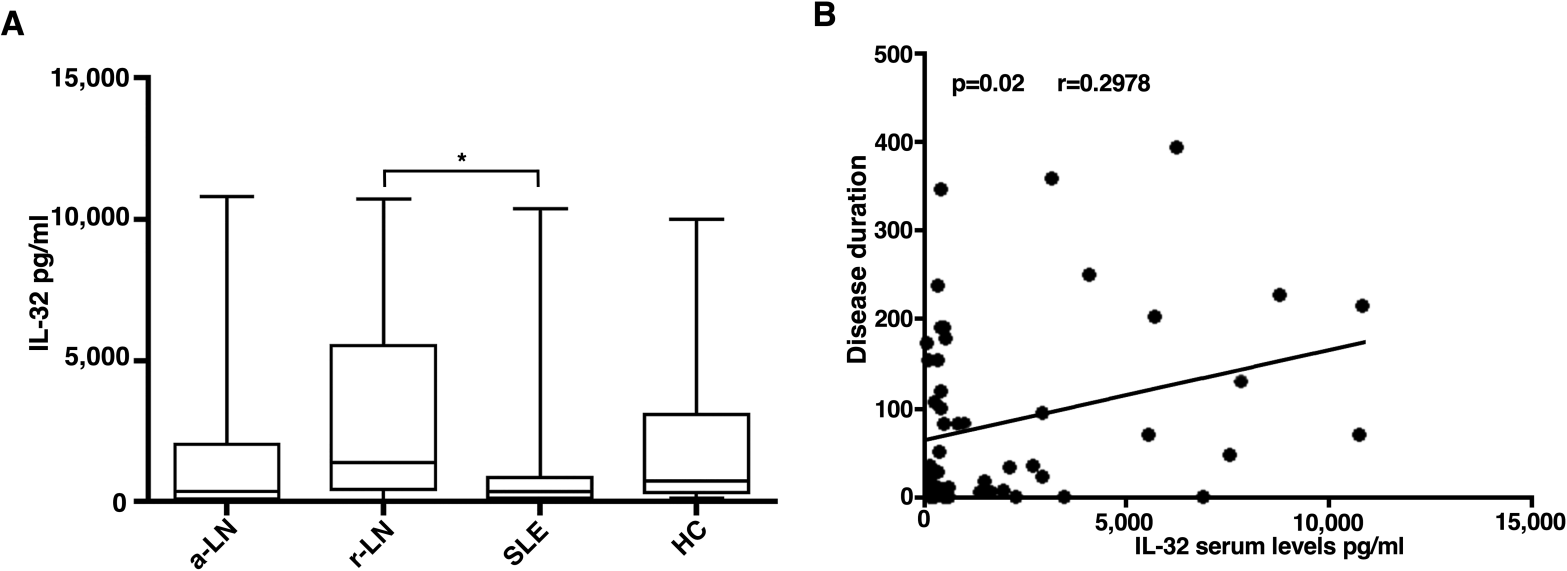
Serum concentration of IL-32 in different study populations. **(A)** IL-32 levels were detected in sera of a-LN (active lupus nephritis), r-LN (remission lupus nephritis) and SLE without renal involvement (Systemic Lupus Erythematosus) patients or healthy donors (HD). *p= 0.03 r-LN *vs* SLE. **(B)** In LN patients, a significant correlation between IL-32 levels (in pg/ml) and disease duration was found. Spearman’s rank correlation coefficient and linear regression are displayed.

IL-32 serum levels were significantly higher in patients with r-LN [median 1368 (3910)] compared to SLE patients without renal involvement [median 203 (662.8)] (p=0.03) (**Figure 1A**). Urinary levels of IL-32 were analysed in 40 LN patients, 20 SLE patients without renal involvement and 20 HC. We didn’t find any significant difference in urinary level of IL-32 through the groups.

We observed a direct correlation between IL-32 serum levels and disease duration in LN patients (p=0.02; r 0.2978) (**Figure 1B**).

### Overexpression of IL-32 in kidney tissue of LN patients

**Figure 2:**
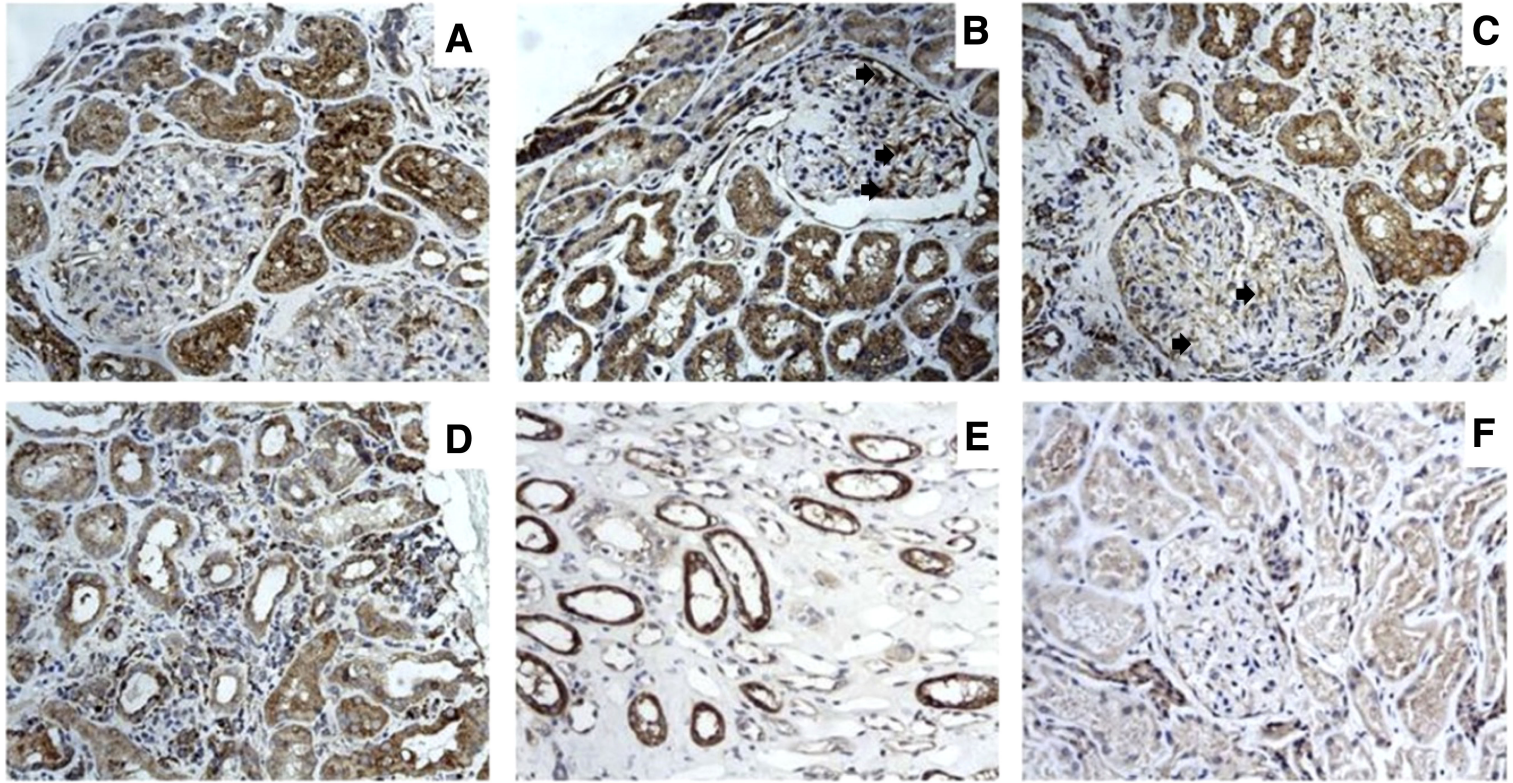
IL-32 expression in renal biopsy samples of patients with LN. Photomicrographs showing 5- μm-thick paraffin embedded sections of renal biopsy specimens obtained from patients with LN **(A–E)** and normal controls **(F)** stained for IL-32. Intense IL-32 expression was observed in epithelial cells of proximal tubular segments of LN patients **(A–C)** and among mesangial cells (B-C, arrows). IL-32-expressing lymphocytes were also observed infiltrating the peri-tubular interstitium **(D)**. Distal tubular expression of IL-32 was also observed in LN patients **(E)**. Representative image showing IL-32 expression in normal control **(F)**.

To evaluate the tissue expression of IL-32 in LN patients, immunohistochemical analysis was performed on renal biopsies of 20 a-LN and 8 HC. Histologically, normal kidney did not exhibit significant immunostaining for IL-32; on the contrary, IL-32 was strongly expressed in renal samples of LN patients, especially in those with class III and IV LN, compared to HC group (**Figure 2**). Intense IL-32 expression was observed in epithelial cells of proximal and distal tubular segments and within mesangial cells; IL-32-expressing lymphocytes were also observed infiltrating the peritubular interstitium (**Figures 2A–D**). The semiquantitative scores for IL-32 in epithelial cells were highly correlated with those for microscopic inflammation on H&E sections (R = 0.57 and p < 0.0001)

### IgG from LN patients induce TBK1 phosphorylation, NF-κB activation and IL-32 expression through a TLR3-dependent mechanism in HEK293/T3 cells

**Figure 3:**
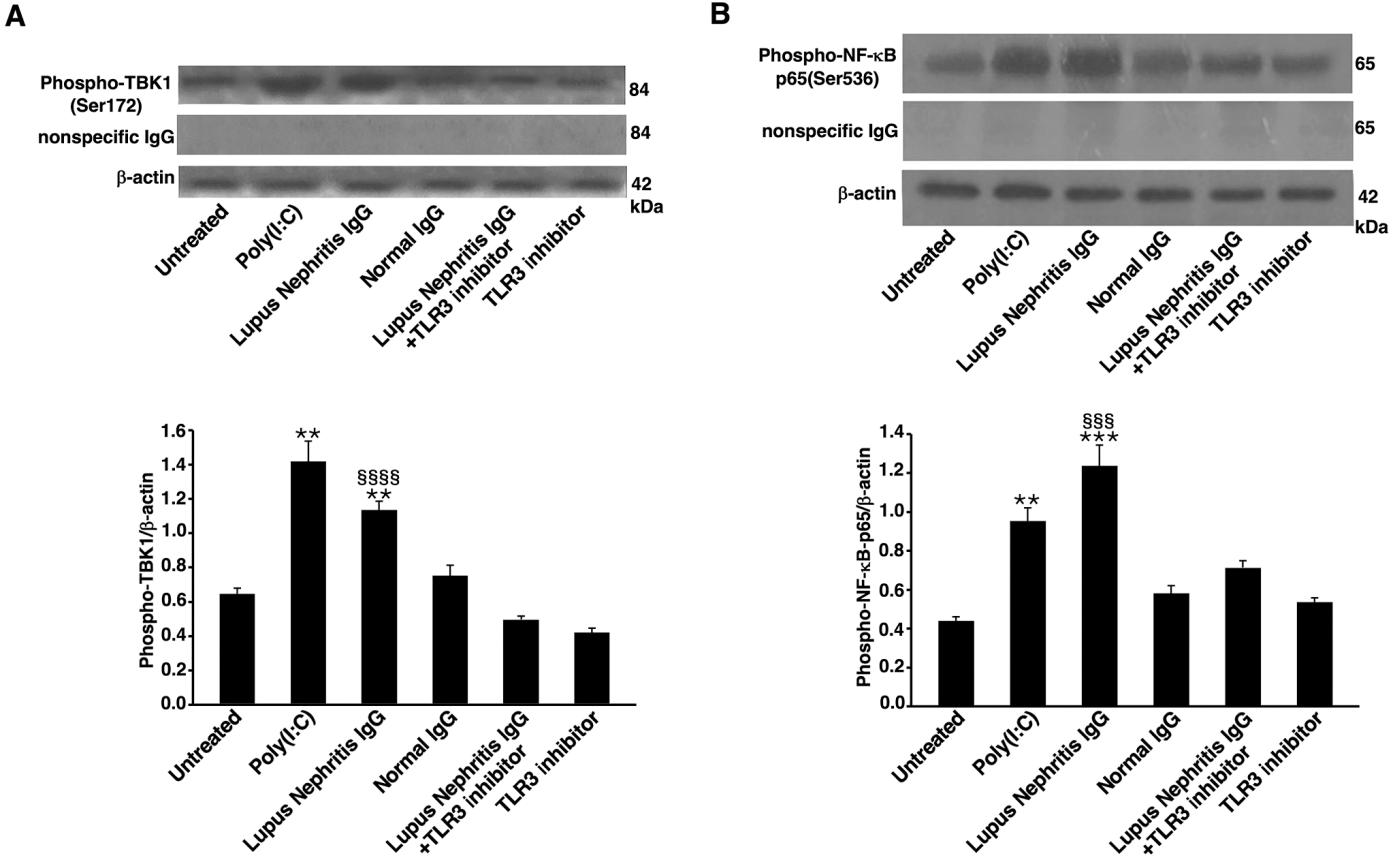
Lupus Nephritis patient IgG induce TBK-1 and NF-κB phosphorylation in HEK293/T3 cells through a TLR3-dependent mechanism. HEK293/T3 cells were treated for 45 min with LN patients IgG (200 μg/ml) and, as controls, with Poly(I:C) (positive control, 10 μg/ml) or NHS-IgG (200 μg/ml). Alternatively, cells were treated with LN patients IgG (200 μg/ml) in combination with a TLR3 inhibitor (27 μM). After treatments cells were analyzed by Western blot for TBK1 phosphorylation and NF-κB-p65 activation. Whole cell extracts were analyzed to determine the levels of: **(A)** phospho-TBK1, using rabbit anti-phospho-TBK1 antibody and, as a control for nonspecific reactivity anti-rabbit IgG. For loading control, anti-β-actin monoclonal antibody was used. Densitometric phospho-TBK1/β-actin ratios are shown. Results represent the mean ± standard deviation (SD) from three independent experiments. Statistical analysis indicates: **p < 0.01 *vs* Untreated; §§§§p < 0.0001 *vs* Lupus Nephritis IgG+TLR3 inhibitor. **(B)** phospho-NF-κB-p65 using rabbit anti-phospho-NF-κB-p65 antibody and, as a control for nonspecific reactivity anti-rabbit IgG. For loading control, anti-β-actin monoclonal antibody was used. Densitometric phospho-NF-κB-p65/β-actin ratios are shown. Results represent the mean ± standard deviation (SD) from three independent experiments. Statistical analysis indicates: **p < 0.01 *vs* Untreated; ***p < 0.001 *vs* Untreated; §§§p < 0.001 *vs* Lupus Nephritis IgG+TLR3 inhibitor.

Analysis, by Western blot, of cell lysates from HEK293/T3 showed that LN-IgG, as well as Poly(I:C), a TLR3 agonist used as positive control, induced TBK1 phosphorylation (**Figure 3A**) and NF-κB activation (**Figure 3B**), as revealed, respectively, by anti-phospho-TBK1/NAK and anti-phospho-NF-κB p65 antibody reactivity. Virtually, no increase in this activation was observed in untreated cells or following NHS-IgG treatment. On the contrary, TBK1 and NF-κB p65 activation significantly decreased when cells were treated with TLR3 inhibitor in combination with LN-IgG. As a control, no significant activation was evident when cells were treated with TLR3 inhibitor alone. The specificity of the antibody reaction was also verified using anti-rabbit IgG and in western blot results no band appeared (**Figure 3**).

**Figure 4:**
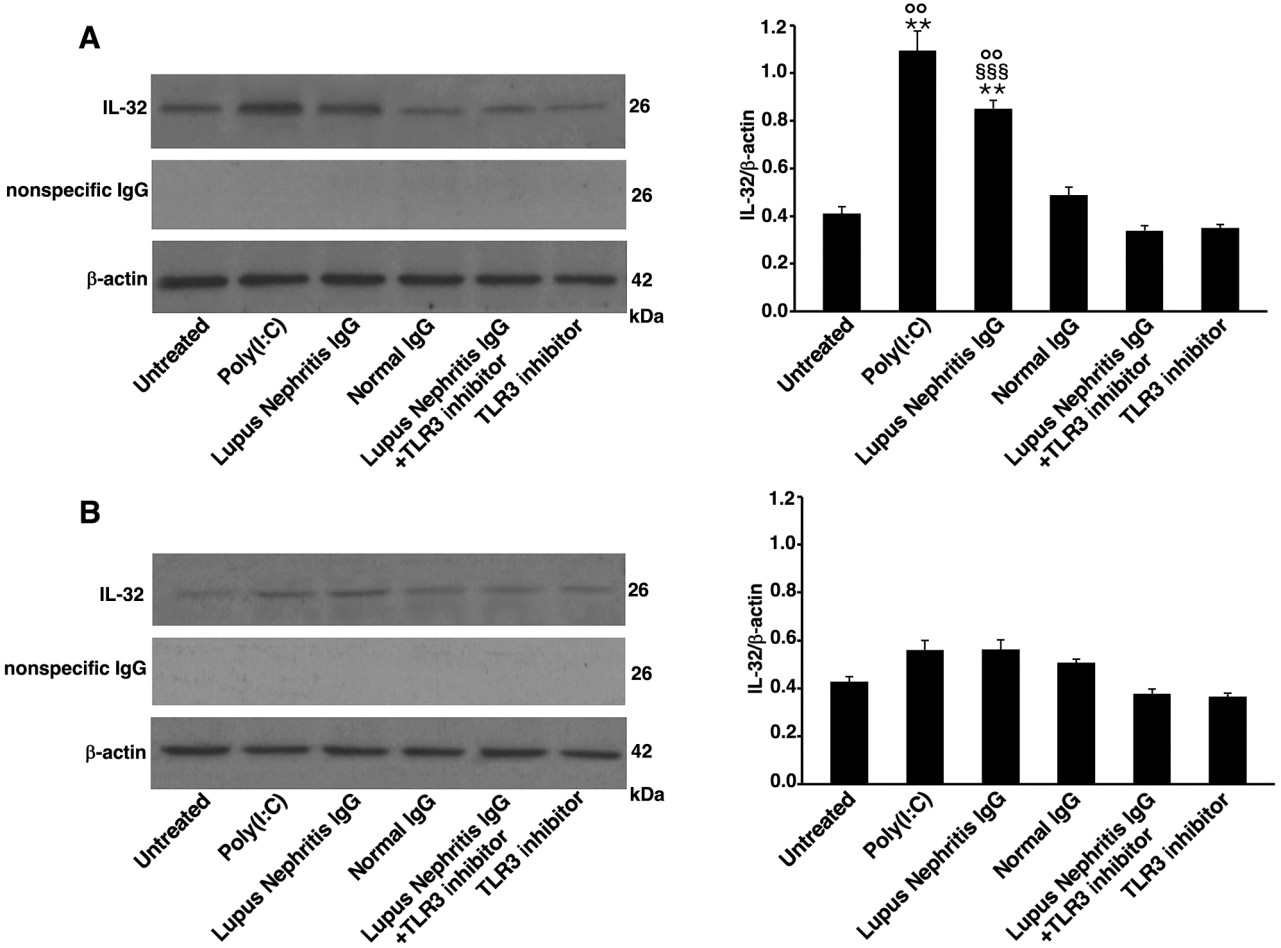
Lupus Nephritis patient IgG increase IL-32 expression in HEK293/T3 cells through a TLR3-dependent mechanism. Cells were treated for 16 h with LN patients IgG (200 μg/ml) and, as controls, with Poly(I:C) (positive control, 10 μg/ml) or NHS-IgG (200 μg/ml). Alternatively, cells were treated with LN patients IgG (200 μg/ml) in combination with a TLR3 inhibitor (27 μM). After treatments, Western blot analysis was performed in **(A)** HEK293/T3 cells, stably transfected with TLR3 gene and **(B)** untransfected HEK293 cells. IL-32 expression was investigated using rabbit anti-IL-32 antibody and, anti-rabbit IgG as a control for nonspecific reactivity. For loading control was used anti-β-actin monoclonal antibody. Densitometric IL-32/β-actin ratios are shown. Results represent the mean ± standard deviation (SD) from three independent experiments. Statistical analysis indicates: **p < 0.01 *vs* Untreated; §§§p < 0.001 *vs* Lupus Nephritis IgG+TLR3 inhibitor; °°p < 0.01 *vs* untransfected HEK293 cells.

To determine whether LN-IgG were able to trigger IL-32 expression in a TLR3-dependent manner, we analyzed, by Western blot, HEK293/T3 and control untransfected HEK293 cells. Results showed that IL-32 expression was significantly increased in HEK293/T3 cells stimulated with LN-IgG compared to untreated cells or treated with NHS-IgG (**Figure 4A**). IL-32 expression induced by LN-IgG was similar to that one triggered by Poly(I:C), a TLR3 agonist used as positive control. The TLR3 inhibitor used in combination with LN-IgG induced a significant reduction of IL-32 expression. The specificity of the antibody reaction was also verified using anti-rabbit IgG and no band was evident observing obtained results (**Figure 4A**). Interestingly, results obtained analyzing untransfected HEK293 cell samples showed a significant lower IL-32 expression (**Figure 4B**). Therefore, these data further support the hypothesis of TLR3 involvement in LN-IgG-induced IL-32 production through activation of TBK1 and NF-κB.

## Discussion

The results of this study demonstrate for the first time that IL-32 is strongly up-regulated in LN patients and that in renal tissue IL-32 expression could be induced through TLR3 activation by the LN patients’ antibodies.

Autoantibodies and immune complexes, deposited at the glomerular level or formed *in situ*, induce cytokine secretion in resident renal cells, which may promote the innate and adaptive immune response; leukocytes infiltrating kidney tissue further release pro-inflammatory cytokines that amplify the immune response and damage progression (4, 29).

IL-32 plays a key role both in innate and adaptive immune responses. Based on different alternative splicing sites, IL-32 has nine isoforms: IL-32α, IL-32β, IL-32γ, IL-32δ, IL-32ϵ, IL-32ζ, IL-32η, IL-32θ, and IL-32small (IL-32sm) (30). Few studies have evaluated IL-32 serum levels in patients with SLE, with contrasting results; none of the previous studies compared IL-32 in SLE patients with and without kidney involvement (31, 32). Inoue and colleagues assessed IL-32γ in 51 SLE patients, and they found detectable levels only in 3, all with kidney involvement (19). Similarly, in a study with larger case series, higher serum levels of IL-32γ were detected in patients with LN than in those without kidney involvement and healthy subjects; interestingly, serum level of IL-32γ positively correlated with renal SLEDAI, histologic activity and chronicity indices of LN (20). Thus, these apparent discrepancies in the literature regarding IL-32 serum levels in patients with SLE may depend on the different selection of patients (with or without kidney involvement). Moreover, it was also demonstrated that IL-32γ is involved in cell death mechanisms, especially apoptosis (33).

In our study, patients were grouped according to the presence or absence of kidney involvement. We demonstrated an increase of IL-32 serum levels in patients with inactive LN compared to SLE patients without renal involvement, while we didn’t find any difference between patients with active or inactive LN. We also found no differences between patients and controls in the urinary levels. These results seem to suggest that IL-32 serum and urinary levels are not useful as biomarkers of disease activity and renal involvement.

We further evaluated IL-32 expression in renal biopsies of patients with active LN and in normal kidney tissue. Our results clearly showed that IL-32 is exclusively expressed by kidney tissue form LN patients mainly in the context of epithelial cells of proximal and distal tubular and in mesangial cells. We also detected IL-32-expressing lymphocytes infiltrating the peritubular interstitium. These findings, together with the correlation found between the semi-quantitative epithelial scores for IL-32 and those for microscopic inflammation on H&E sections, may suggest a pathogenic role of IL-32 in LN.

IL-32 is involved in the production of Th1- and Th17-polarizing cytokines via a PLC/JNK/NF-kB-dependent pathway. Moreover, in LN IL-32 overexpression could be the result of innate immune system activation (34). In this regard, TLR3 pathway has been previously suggested to be involved in the pathogenesis of LN (12) and an increased expression of IL-32 following TLR3 activation has been already demonstrated in epithelial cells (13) and fibroblast-like synoviocytes (14). To explore the link between IL-32 and TLR3 pathway in LN disease, we demonstrated the ability of IgG from SLE patients to activate a typical TLR3 signal transduction, relating to TBK1 and NF-κB activation (35), and to increase IL-32 production, in a human embryogenic renal cell line transfected with TLR3. Thus, with the aim of studying the stimulation of human TLR3, we selected HEK293/T3 cells for use in our *in vitro* experiments to verify the involvement of TLR3 signal transduction pathway in IL-32 production. Taken together, these data may confirm a role of TLR3 pathway in the production of IL-32. Further studies are in progress to better clarify the signalling pathway involved in IL-32 activity in order to identify new molecular therapeutic targets in patients with LN.

In conclusion, our results do not support IL-32 as a serological o urinary biomarker of kidney involvement. Conversely, the increased expression of IL-32 in renal biopsies of LN patients, coupled with IL-32 production induced in renal cells in a TLR3-dependent manner, after *in vitro* stimulation with IgG from patients, suggest a local production of this cytokine. Furthermore, the strong correlation between IL-32 in epithelial cells and inflammation in renal biopsy samples suggests a possible role for IL-32 in the pathogenesis of LN.

## Data Availability

The raw data supporting the conclusions of this article will be made available by the authors, without undue reservation.
